# RAS‐targeted cancer therapy: Advances in drugging specific mutations

**DOI:** 10.1002/mco2.285

**Published:** 2023-05-27

**Authors:** Cen Liu, Danyang Ye, Hongliu Yang, Xu Chen, Zhijun Su, Xia Li, Mei Ding, Yonggang Liu

**Affiliations:** ^1^ Beijing University of Chinese Medicine Beijing China; ^2^ Institute of Genetics and Developmental Biology Chinese Academy of Sciences Beijing China

**Keywords:** allelic RAS mutation, cancer, personalized therapy, RAS inhibitor

## Abstract

Rat sarcoma (RAS), as a frequently mutated oncogene, has been studied as an attractive target for treating RAS‐driven cancers for over four decades. However, it is until the recent success of kirsten‐RAS (KRAS)^G12C^ inhibitor that RAS gets rid of the title “undruggable”. It is worth noting that the therapeutic effect of KRAS^G12C^ inhibitors on different RAS allelic mutations or even different cancers with KRAS^G12C^ varies significantly. Thus, deep understanding of the characteristics of each allelic RAS mutation will be a prerequisite for developing new RAS inhibitors. In this review, the structural and biochemical features of different RAS mutations are summarized and compared. Besides, the pathological characteristics and treatment responses of different cancers carrying RAS mutations are listed based on clinical reports. In addition, the development of RAS inhibitors, either direct or indirect, that target the downstream components in RAS pathway is summarized as well. Hopefully, this review will broaden our knowledge on RAS‐targeting strategies and trigger more intensive studies on exploiting new RAS allele‐specific inhibitors.

## Graphical abstract

Personalized therapeutic strategy targeting specific mut‐RAS‐induced cancers is essential. Cancer type, RAS mutation type, activation of downstream pathways, and clinicopathological background should be comprehensively considered when designing strategies for drug combinations.

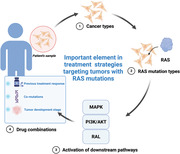

## 1 INTRODUCTION

Rat sarcoma (RAS) proteins belong to the small‐molecule G protein family. As binary molecular switch, RAS GTPases cycle between guanosine triphosphate (GTP)‐bound active form and guanosine diphosphate (GDP)‐bound inactive form, facilitating “on” or “off” state in signal transduction. There are three canonical *RAS* genes, kirsten‐RAS (*KRAS*), neuroblastoma‐RAS (*NRAS*), and harvey‐RAS (*HRAS*), encoding four RAS proteins (KRAS4A, KRAS4B, NRAS, and HRAS). Among *RAS* genes, *KRAS* could encode two proteins (KRAS4A and KRAS4B) due to alternative RNA splicing. RAS and its downstream pathways control a variety of activities, such as cell proliferation, survival, migration, etc.^1^

Since the discovery of mutated oncogenic RAS in human cancer in 1982, it became a common sense that RAS mainly drives the formation and development of tumor through point mutations.^2^, ^3^, ^4^ Oncogenic RAS family mutations have been found in about 27% of all human cancers^5^, ^6^, ^7^, ^8^, ^9^; thus, intensive research effort has been made to study RAS protein structure and biochemistry and develop anti‐mutated RAS drugs for cancer therapy.^10^ However, the study is full of failures and obstacles for the following reasons: (1) RAS protein structure is smooth globular with shallow depressions, which lack well‐defined hydrophobic pockets for targeting study. (2) As small GTPases, there are also studies that attempt to competitively inhibit RAS binding to GTP, based on the fact that small G protein RAS needs to bind to GTP to form an activated form. However, there is picomolar binding strength between RAS and GTP, and the concentration of GTP in the cytoplasm reaches ∼0.5 mmol/L.^11^, ^12^, ^13^, ^14^ Therefore, the development of competitive GTP‐binding inhibitors is almost impossible. Thus, the development of oncogenic RAS inhibitors had been stuck in bottleneck until recent success in specific KRAS^G12C^ inhibitors.^15^, ^16^, ^17^, ^18^

Subsequent clinical data manifest that KRAS^G12C^ inhibitor treatment efficacy differs in cancers with different oncogenic RAS mutations or even in different cancer types with KRAS^G12C^ mutation. Therefore, knowledge of the molecular structural property, biochemistry, and biology will lay a foundation for designing an allelic‐specific mutated RAS inhibitor for personalized cancer treatment.

Based on the mutation sites and important domains affecting RAS function, this review summarizes the structural biochemical differences of different mutations, so as to provide reference for the development of inhibitors targeting specific mutations. Furthermore, in order to achieve better clinical treatment with inhibitors, the review also lists the clinicopathological features among cancers commonly carrying RAS mutations and responses to treatment strategies, providing reference for the appropriate selection of personalized treatment for cancers with RAS mutations.

## 2 PHYSIOLOGICAL ACTIVITIES OF FUNCTIONAL RAS DOMAINS

RAS is a member of the small‐molecule G protein family that is activated by GTP binding. Different RAS paralogs share similar structural compositions: a highly conserved G domain (aa 1–165) and a hypervariable region (aa 166–178/179).^19^, ^20^, ^21^

The G domain is composed of an effector lobe (aa 1–86) and an allosteric lobe (87–165).^22^ The former contains two switch regions: switch I and II, along with a P‐loop.^23^, ^24^, ^25^ The latter contains regulatory sites that result in conformational change in switch II once it binds calcium or acetate.^23^

**FIGURE 1:**
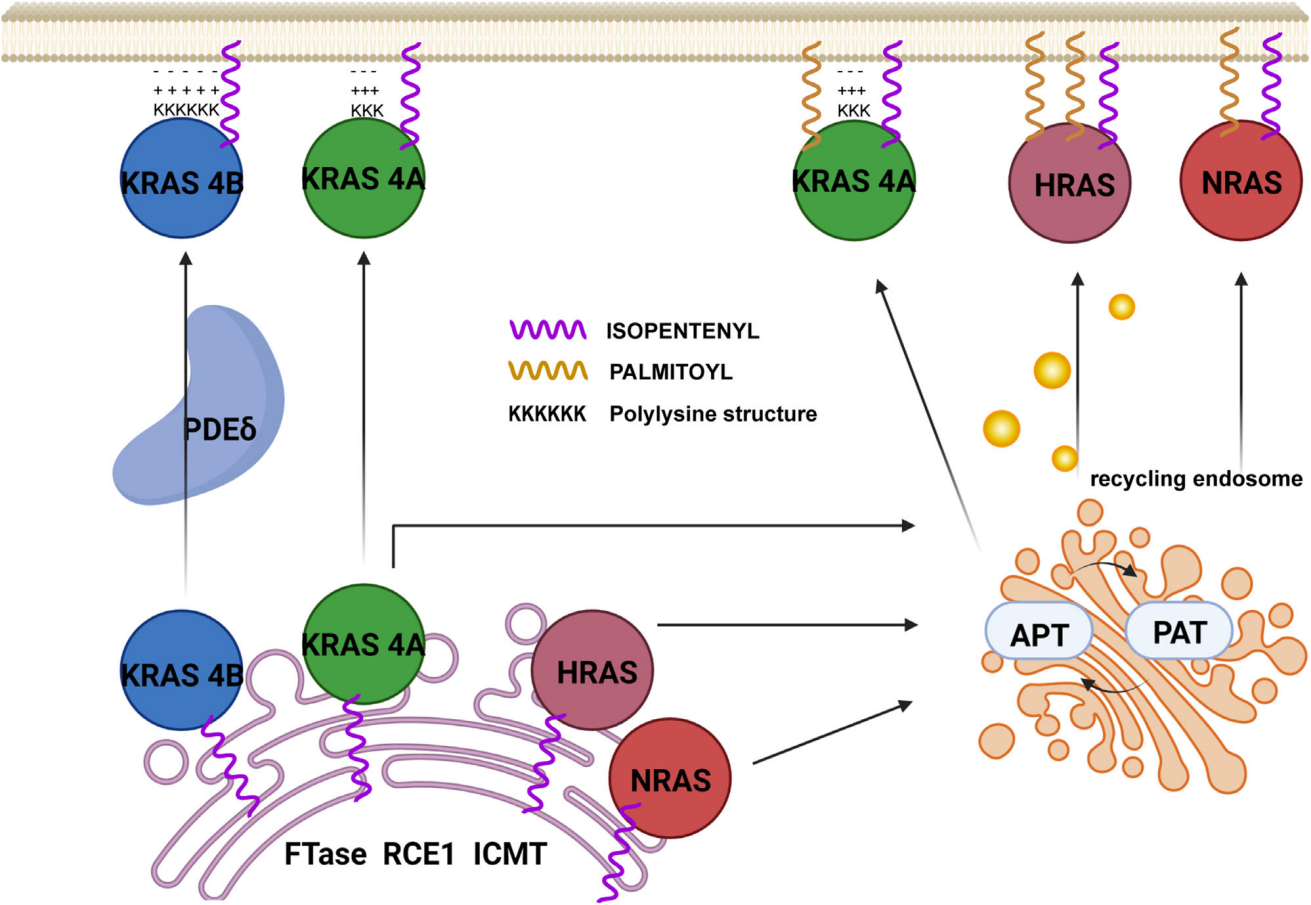
The process of Rat sarcoma (RAS) binding to plasma membrane after post‐translational modification. CAAX motifs at C‐terminal of RAS, consisting of cysteine, aliphatic amino acids, and a variable amino acid, help RAS localize to specific plasma membrane microdomains and subsequently pass signals to the downstream.^30^ The post‐translational modification of CAAX is achieved by farnesylation, hydrolysis by RAS and a‐factor converting enzyme 1 (RCE1) and carboxymethylation by prenylcysteine carboxyl methyltransferase (pcCMT), followed by palmitoylation by palmitoyltransferases (PAT) in Golgi (harvey‐RAS (HRAS), neuroblastoma‐RAS (NRAS), KRAS4A). The difference between KRAS4B and other paralogs is that the CAAX of KRAS4B is able to bind to membrane depending on lysine residues.^31^, ^32^ The figure was made using Biorender.

Before playing a role, the C‐terminal CAAX box of RAS (comprising cysteine (C) and aliphatic (a) and variable (X) amino acids) (CAAX) tail of RAS needs to undergo a series of modifications.^26^, ^27^, ^28^ As shown in Figure 1, after being modified by several enzymes, such as RAS and a‐factor converting enzyme 1 (RCE1), prenylcysteine carboxyl methyltransferase (pcCMT), and palmitoyltransferases (PAT), CAAX is able to associate with the cytoplasmic leaflet of cellular membrane, and then RAS is recruited to the plasma membrane and activated by receptor tyrosine kinase (RTK) signals and subsequently recruits downstream effector molecules to achieve signal transduction.^29^, ^30^, ^31^, ^32^, ^33^ In the process, the cysteine in the CAAX tail is first linked to a farnesyl group (15‐carbon) via the thioether bond by FTase. Alternatively, the cysteine of CAAX would also be modified with a geranyl group (20‐carbon) by geranylgeranyltransferase I (GGTase I) if FTase was blocked by inhibitors. RCE1 then excises the AAX amino acids by recognizing farnesyl cysteine and conjugates cysteine with a carboxyl group. The carboxyl group is subsequently modified by pcCMT to produce carbomethoxy, which increases the lipophilicity of RAS and helps RAS bind to the membrane. After this, the modification processes of different paralogs begin to distinguish each other. In addition to farnesyl cysteine, the remaining cysteine residues of NRAS and HRAS would be palmitoylated by PAT, which is located in the Golgi to facilitate their transport to membrane. Unlike the former, KRAS4B will not be modified by PAT because there is no remaining cysteine residue available. However, the polybase region of KRAS4B will bind membrane through electrostatic interaction. In addition, KRAS4A has both a discontinuous polybase region and excess cysteine available for palmitoylation, which help KRAS4A choose whether or not to be modified with PAT.^30^

After binding to the plasma membrane, the RAS protein would be activated by the upstream RTK signal and then converted into an active form for downstream signaling. RAS activity switches between RAS–GTP (activated) and RAS–GDP (inactive) binding forms, which is regulated by guanine nucleotide exchange factors (GEFs) and GTPase‐activating proteins (GAPs). Recently, however, RAS has been found to be activated by cytoplasmic RTK granules rather than RTKs located in the plasma membrane. Tulpule et al.^34^ found that oncogenic RTKs, losing their lipid membrane‐targeting sequences, could form membrane‐less cytoplasmic protein granules, in which the RTK granules activate RAS in a lipid membrane‐independent manner. Besides, RAS activity can be regulated by other modifications, such as phosphorylation,^35^, ^36^, ^37^ ubiquitination,^38^, ^39^, ^40^ lysine acetylation,^41^, ^42^, ^43^ and lysine methylation.^44^

In cells, once an upstream signal is received, GEF (such as SOS) forms a complex with RAS binding to GDP while promoting the dissociation of GDP from RAS. Then, GTP would replace GEF in the complex to form the active GTP‐bound RAS to pass the signal downstream.^45^, ^46^ Normally, the activation of RAS signaling pathway is transient under physiological conditions. After the signal transmission is complete, GAP could boost RAS intrinsic GTPase activity to dissociate from GTP and restore it to GDP‐bound form, thereby closing related signaling pathways.^47^ However, when a specific point mutation of RAS occurs, the intrinsic GTPase activity or GAP‐binding ability of RAS would be attenuated. Thus, the existence duration of RAS–GTP, along with the activation duration of the downstream signaling pathway, is prolonged, resulting in abnormal cell proliferation as well as tumor occurrence and development.^48^, ^49^, ^50^, ^51^

## 3 GENERAL DAMAGE INDUCED BY RAS MUTATIONS AT HOT‐SPOT SITES

**FIGURE 2:**
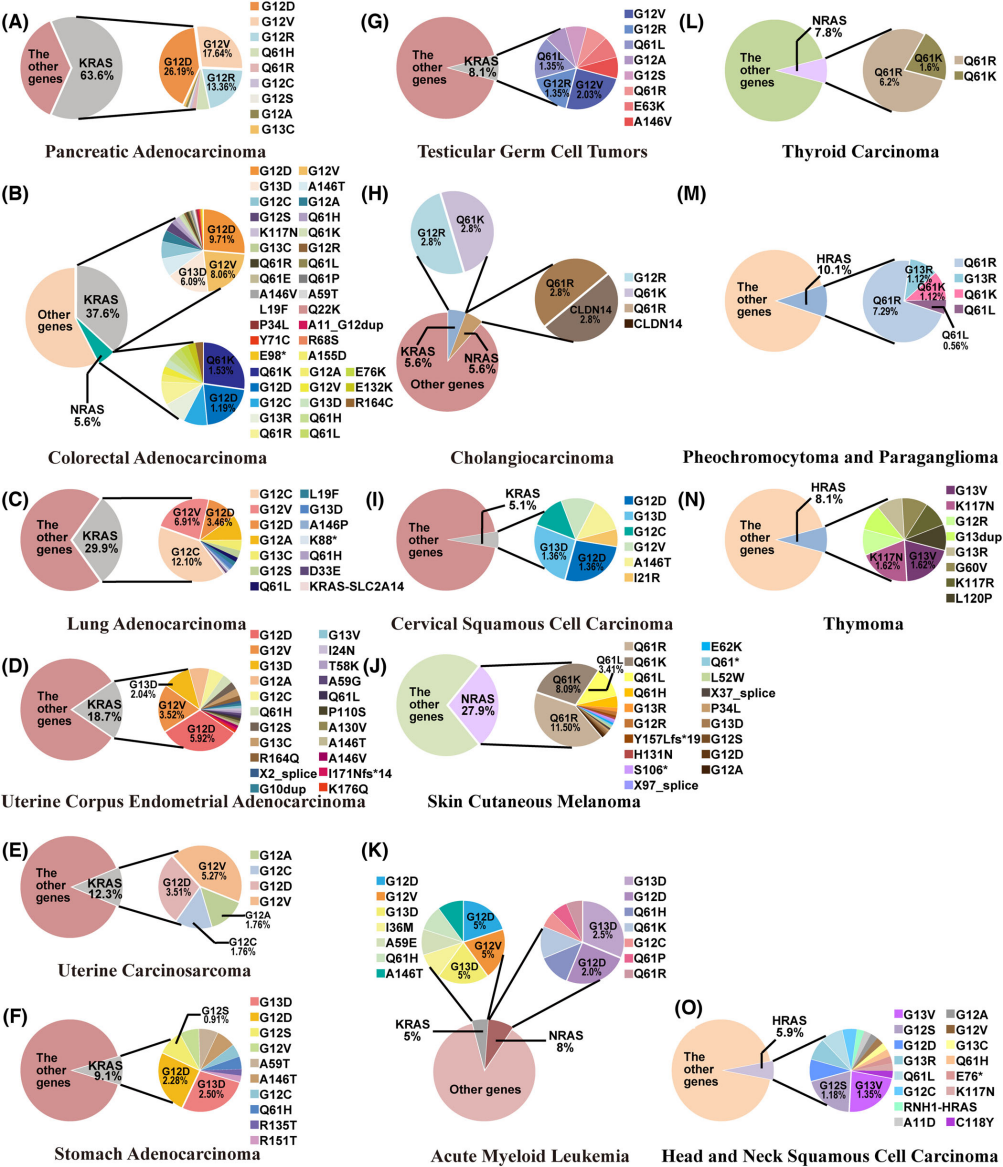
The frequency of RAS mutations in human cancers. The data come from TCGA PanCancer Atlas Studies. The activating mutations of RAS occur predominantly at codons 12, 13, and 61 and kirsten‐RAS (KRAS) has the most tendency to be mutated in the three paralogs. Clinically, RAS mutations are most prevalent in pancreatic adenocarcinoma (PC) (70%), colorectal cancer (CRC) (40%), and non‐small cell lung cancer (NSCLC) (30%). (A) PC; (B) colorectal adenocarcinoma; (C) lung adenocarcinoma; (D) uterine corpus endometrial adenocarcinoma; (E) uterine carcinosarcoma; (F) stomach adenocarcinoma; (G) testicular germ cell tumors; (H) cholangiocarcinoma; (I) cervical squamous cell carcinoma; (J) skin cutaneous melanoma; (K) acute myeloid leukemia; (L) thyroid carcinoma; (M) pheochromocytoma and paraganglioma; (N) thymoma.

There are mainly three *RAS* paralogs with different mutation frequencies in cancer, including *KRAS* (85%), *NRAS* (11%), and *HRAS* (3%).^52^, ^53^ When oncogenic amino acid substitution mutations occur, changes in RAS protein structure and function lead to persistently activated downstream signaling pathways. Clinically, particular isoform, mutation frequency, and amino acid substitution preference at each site complicate RAS mutation occurrence in various cancers.^54^ The types of RAS mutations and their frequency in related cancers are shown in Figure 2.

RAS mutations occur with high frequency at G12, G13, and Q61,^55^, ^56^, ^57^ causing unique changes in RAS domain conformation^58^, ^59^ and facilitating persistence of RAS–GTP‐binding form.^54^ Extensive studies had been conducted on the structural and functional influences of these special sites on RAS, such as GDP/GTP binding, intrinsic GTPase activity, and effector interaction. The biochemical function of each hot‐spot mutation site and the damage to physiological activities of RAS caused by site‐related mutations are summarized below, including G12, G13, and Q61.

### 3.1 GTP hydrolysis rate

RAS forms a transition state during either GAP‐mediated or RAS‐intrinsic hydrolysis for GTP.^60^, ^61^, ^62^, ^63^ Although the transition state form during RAS–GTP hydrolysis is still controversial,^64^ current studies support that G12, G13 (located in the P‐loop), and Q61 (located in the switch II region) once mutated, the transition state form would be disturbed to some extent, impairing the ability of RAS to hydrolyze GTP. This is likely to be one of the reasons for the oncogenic properties of mutated RAS. The related mechanisms of GTP hydrolysis rate changes caused by G12 and G13 mutations are complicated.

In the normal GAP‐mediated hydrolysis, Arg789 (also known as arginine finger) of GAP interacts with the carbon atom in the main chain at G12 of RAS.^65^, ^66^, ^67^, ^68^ However, when G12 is mutated to other amino acids, the extra side chains of alternative conflict with the interaction, which stops the formation of transition state complex of G12 mutant and GAP, resulting in a lower rate of the GAP‐dependent hydrolysis of GTP compared to wild type.^69^, ^70^ Similarly, G13 mutants with large side chains are unable to form transition state complexes with GAP, while the mutants with small side chains are able to form transition states.^69^ In addition, there are data showing that mutations in G12 and G13 (G13D) affect the interaction between hydrolysis catalytic residues Q61 of RAS, R789 of GAP and GTP, thereby weakening GAP‐mediated GTP hydrolysis.^59^, ^71^ Another report mentions that the G13V mutant could hinder fast hydrolytic steps by increasing the flexibility of the region of RAS binding to GTP γ‐phosphate group, catalytic water and Arg789.^72^ Surprisingly, Rabara et al.^73^ found that KRAS mutation at G13 is sensitive to GTP hydrolysis stimulated by NF1 (a GAP), which is different from G12 and Q61.

The intrinsic RAS GTPase activity, relative to GAP‐dependent GTP hydrolysis activity,^74^ may determine the interaction duration between RAS and downstream objective response rate (RAF)‐related pathways.^19^ The affinity of RAF for KRAS is higher than that of P120GAP and NF1, and more importantly, their binding protein domains of RAS overlap.^75^ At the same time, these differences in affinities may also be one of the reasons for biological behavior differences among mutant forms.^76^ When RAS binds to RAF–ras binding domain (RBD), the combination of Y32 residue located in switch I of RAS and Q61 residue interacts with the γ‐phosphate group in GTP through a bridged water molecule to form a transition state complex.^77^, ^78^ Although the model of specific transition state formation has not yet been determined, it is found that once other amino acids, such as alanine and valine, replace glycine at 12‐position, the steric hindrance caused by oversized side chain would hinder the formation of transition states or reduce the stability.^79^ Moreover, according to previous reports, G13 is also involved in the hydrogen bond interaction in GTP hydrolysis, and the large side chain due to mutation would cause instability of the transition state.^59^

Compared with G12 and G13, Q61 plays a direct and important role in both intrinsic and GAP‐mediated GTP hydrolysis. Q61 regulates nucleophilic water molecules close to the γ‐phosphate of GTP, and the intrinsic hydrolysis rate of RAS significantly reduces once Q61 is mutated.^19^, ^80^ Thus, the hydrolysis rates in Q61 mutants are the lowest.^76^ However, unlike the G12 mutations, the hydrolysis rate of the Q61 mutations could be significantly increased by GAP,^81^ although the mechanism is still unclear.

### 3.2 Nucleotide exchange rate

Compared with wild type, G12 and Q61 mutated RAS, the nucleotide exchange rate in the RAS^G13D^ is significantly higher, and it is more important that this rapid nucleotide exchange is spontaneous through an SOS‐independent way.^81^ Chen et al.^82^ G13D mutation causes the destabilization of the hydrogen bond formed between D30 in SWI switch and GDP, facilitating RAS–GDP to GTP transformation. Additionally, an increase in the structural flexibility of the switch domains SW1 and SW2 in mutant may result in a decrease in the stability of the nucleotide‐binding pocket. For the reasons described above, nucleotide exchange with a rapid rate starts up. It is found that the G13D mutation is rare in HRAS and NRAS compared with other mutations, and there is a speculation that KRAS may have a specific residue background different from the other paralogs to support the instability of the G13D active site.^83^ Unfortunately, there are few data for other types of mutations at G13.

As for G12 and Q61 mutations, Smith et al.^81^ found that without SOS1 mediation, the nucleotide exchange rate of G12V is 1.8 times slower than that of wild type, while the exchange rate of Q61L is 2.4 times faster, and the former requires more SOS1 assistance to reach the wild‐type exchange rate.

### 3.3 Affinity differences in downstream effectors

When mutated, the binding affinity of RAS to different downstream effectors changes, which means that the biases for downstream signaling may change.

There are data showing that the affinities of ARAF and BRAF to wild‐type RAS are stronger than the affinity of G12V mutant, while the binding preference of RGL1 and RALGDS (two GEFs of RAL GTPases) for RAS^G12V^ increases.^84^ In addition, compared to KRAS^WT^‐overexpressing cells, the Akt signaling of HBECsiP53 cells with KRAS^G12C^ overexpression is lower and Ral signaling and anchorage‐independent growth are stronger, whereas the phosphorylated Akt levels are higher and Ral activation is lower when KRAS^G12D^ is overexpressed.^85^

In conclusion, signal transduction bias and variation in downstream signal stringency arising from different RAS mutations suggest that it is necessary to selectively use inhibitors targeting mutation‐preferred pathways in facing different mutations in clinical treatment.

## 4 CHARACTERISTICS OF SPECIFIC RAS MUTATIONS

Although the previous section shows the general impact of RAS mutations associated with respective hot‐spot sites, each mutation has specific biochemical characteristics. For example, the development of G12C inhibitor is based on the feature that G12C is in the GDP‐binding form for a longer time than KRAS^WT^ or other mutations.^86^ Therefore, we introduce the biochemical characteristics of common oncogenic RAS mutations in this section. In addition, in order to emphasize the significance of specific mutation, pathological differences brought by different mutations, such as the tendency of co‐mutations, transformation capacity, and clinicopathological features, are also listed.

### 4.1 Differences in structure and biochemical signature

The function of protein depends on its structure. Table 1 summarizes the structural differences of the following RAS mutations, expecting to be helpful for the design of allele‐specific inhibitors.

**TABLE 1:** Structural differences of RAS mutations.

Allele	Difference	Potential biochemical impact	References
KRAS^G12C^	Cysteine insertion may improve the electrostatic environment around γ‐phosphate of GTP	Intrinsic hydrolysis rate close to wild type	^76^
	More exposed nucleotide binding site	An increased propensity for the conformational transition of KRAS from inactive to active	^59^
	R789 of GAP away from GTP in KRAS^G12C^–GTP–GAP complex	A decrease in the rate of GAP‐mediated hydrolysis	^59^, ^76^
	Broken hydrogen bond between Q61 and the γ‐phosphate of GTP		
KRAS^G12D^	The atom OE1 of Q61 side chain away from GTP in KRAS^G12D^–GTP–GAP complex	A decrease in the rate of GAP‐mediated hydrolysis	^59^
	More exposed nucleotide‐binding site	An increased propensity for the conformational transition of KRAS from inactive to active	^59^
	Deviation of SII residues toward the α3 helix and disruption of the hydrogen bonding network in SII	Slow GTP hydrolysis rate	^87^
	The electrostatic repulsion between the carboxylate group on the side chain of aspartic acid and the γ‐phosphate of GTP	The binding force of KRAS^G12D^ to GppNp is weaker than that of KRAS^G12V^ or KRAS^WT^	^88^
KRAS^G12V^	The atom OE1 of Q61 side chain away from GTP in KRAS^G12V^–GTP–GAP complex	A decrease in the rate of GAP‐mediated hydrolysis	^59^
KRAS^G12R^	The flexibility of Q61 affected and the cooperation of Q61 with nucleophilic water broken	A decrease in GAP‐dependent hydrolysis rate	^76^
	The side chain of arginine creates a steric conflict with the Y32 residue, and displaces adjacent coordinated water	A decrease in intrinsic hydrolysis rate	^76^
KRAS^G12A^	Alanine insertion may change the electrostatic environment around the γ‐phosphate of GTP and reorder the solvent	A decrease in intrinsic hydrolysis rate	^76^
KRAS^G13D^	The arginine finger of GAP is blocked from accessing phosphate of GTP	A decrease in GAP‐dependent hydrolysis rate	^73^
KRAS^G13R^	The arginine finger of GAP is blocked from accessing phosphate of GTP	A decrease in GAP‐dependent hydrolysis rate	^73^
KRAS^Q61H^	The atom ND1 of H61 side chain away from GTP in KRAS^G12V^–GTP–GAP complex	A decrease in the rate of GAP‐mediated hydrolysis	^59^
HRAS^Q61L^	An increase in the flexibility of the SII domain, while in the opposite with RAF–RBD binding	Reduced intrinsic hydrolysis rate and stronger RAF/MEK/ERK signal	^89^, ^90^

Abbreviations: GTP, guanosine triphosphate; HRAS, harvey‐RAS; KRAS, kirsten‐RAS; NRAS, neuroblastoma‐RAS; RAF, Serine/threonine‐protein kinase RAF; RAS, rat sarcoma; RBD, RAS binding domain.

By comparing various biochemical indicators of KRAS mutants, Hunter et al.^76^ found that small structural differences due to mutation led to specific biochemical signatures. For example, alteration in nucleotide exchange rate is most notable in KRAS^G13D^. According to Table 1, KRAS^G13D^ enables faster nucleotide exchange independent of GEFs. As for the intrinsic hydrolysis rate, KRAS^G12A/G12R/Q61H/Q61L^ mutation brings about a drop to the lowest level.^76^ On the contrary, unlike other mutants with severely impaired hydrolysis capacity, KRAS^G12C^ has no significant effect on intrinsic hydrolysis rate compared to wild type.^76^ Alternatively, it is more likely to develop the GDP‐binding form KRAS^G12C^ than other mutations, which gives opportunities for the generation of direct inhibitors.

Overall, biochemical differences among RAS mutations offer an opportunity to design specific inhibitors of RAS mutants.

### 4.2 Differences in co‐mutation

Rabara et al.^73^ reported that the KRAS^G13X^ is more sensitive to GTP hydrolysis stimulated by NF1 (a GAP) than G12 mutants and Q61 mutants and partially dependent on upstream signaling from epidermal growth factor receptor (EGFR) or other RTK, which may enable EGFR inhibitors to prevent the development of colorectal cancer (CRC) with G13D mutation.^91^ In addition, Rabara et al.^73^ also pointed out that cells with low‐frequency and weakly oncogenic KRAS mutations are more prone to additional mutations. In other words, their genomes have higher genetic instability. For example, KRAS^G13X^ and NRAS^A146T^ are occasionally associated with NF1 mutations, while NF1 mutations are rarely observed in cells with KRAS^G12X^ and KRAS^Q61X^ mutations. According to the above, EGFR inhibitors may be an option that is worth considering in the treatment of cancers with KRAS^G13x^ and normal NF1 activity.

### 4.3 Differences in transformation capacity

The transformation capacities of different mutants vary. By comparing a large number of c‐Ha‐ras1 mutants, Seeburg et al.^92^ showed that the transformation abilities of HRAS^G12R^ and HRAS^G12V^ mutations are stronger than those of other mutations at codon 12. Additionally, by transfecting NIH3T3 cells with plasmids expressing wild‐type or mutated KRAS, Smith et al.^93^ observed that the transformation ability of mutations at codon 12 was slightly stronger than that of mutations at codons 13, 61, 146, and 117.

### 4.4 Clinicopathological varieties in different RAS mutations

In addition to the above, the clinicopathologic features of cancers carrying different RAS mutations are also different. Table 2 summarizes the pathological differences in various RAS mutation‐induced cancers.

**TABLE 2:** Clinicopathological differences among cancers.

Cancer	Mutation	Difference	References
PC	KRAS^G12D^	Lower survival rate and poorer prognosis after first‐line gemcitabine therapy and shorter OS in patients with locally advanced and/or metastatic PDAC	^94^
		Little response to the combination of cobimetinib and gemcitabine in PDAC	^95^
		A strong association with early distant metastasis after radical tumor resection and shorter postoperative OS and PFS in PDAC	^96^
	KRAS^G12V^	Little response to the combination of cobimetinib and gemcitabine in PDAC	^95^
CRC	KRAS^G12D^	Poor response to FOLFOX treatment and high risk of disease recurrence	^97^
		Shorter OS compared with patients with KRAS^WT^	^98^
	KRAS^G12X^	For patients with CRLM undergoing radical liver resection, KRAS G12 mutations is associated with shorter OS than G13 mutations, especially G12V or G12S. Besides, G12D and G12V are related to shorter OS and PFS	^99^, ^100^
	KRAS^G12V^	A higher risk of relapse and death	^88^, ^101^
		Poorer survival in BRAF^WT^ CRC	^102^
	KRAS^G13D^	Multiple metastatic sites as the disease progressed	^103^
		A tendency to lead lymph node metastasis, more common in advanced cancer and associated with higher PFS	^104^
	KRAS^G12X^	Associated with mucus histotype and favoring signaling pathways involved in regulating mucin production in colonic mucosal cells	^104^
	KRAS^G12C^	Shorter OS, higher basal EGFR activation, and reduced immune profile	^105^
NSCLC	KRAS^G12C^	Shorter PFS treated with PD‐L1 inhibitors among patients with high PD‐L1 expression	^106^
	KRAS^G12V^	Worse OS, PFS, and recurrence rates	^107^
		Shorter survival, shorter duration of response to initial chemotherapy, and shorter OS after immunotherapy in patients with advanced NSCLC with KEAP1/NFE2L2 co‐mutation	^108^
		More pleural–pericardial metastases after thoracic surgery compared with other mutations	^107^

Abbreviations: CRC, colorectal cancer; CRLM, colorectal liver metastases; EGFR, epidermal growth factor receptor; NSCLC, non‐small cell lung cancer; OS, overall survival; PC, pancreatic adenocarcinoma; PDAC, pancreatic ductal carcinoma; PD‐L1, programmed death‐1‐ligand 1; PFS, progression‐free survival.

## 5 DEVELOPMENT OF DIRECT MUT‐RAS INHIBITORS

### 5.1 Non‐mutation‐specific inhibition ways targeting oncogenic RAS

The structure of RAS has been studied in depth. However, it was not until recent years that inhibitors that bind directly and block the abnormal activities of oncogenic RAS were developed. The first hurdle in targeting RAS mutations is the lack of deep binding pockets. RAS is similar to globular protein with smooth surface and shallow depressions, lacking a clear hydrophobic pocket and obscuring allosteric site.^109^ The other strategy is reducing the active state of RAS–GTP by competing for the GTP‐binding region. However, the binding strength of RAS with GTP reaches picomolar level and the concentration of GTP in the cytoplasm reaches as high as ∼0.5 mmol/L; thus, it is too difficult to develop drugs that compete with RAS–GTP binding.^11^, ^59^

After decades of efforts, some “hidden weaknesses” of RAS have gradually been uncovered. Lu et al.^110^ found that the formation of activated RAS experiences multiple sub‐states, and there is a new allosteric druggable site P4 hidden in the protein, which could regulate the GTPase activity of RAS. More importantly, this site is located at the interface of the RAS domain, which interacts with downstream effectors, so targeting the interaction of RAS and downstream effectors becomes a new strategy to design drugs. In addition, some research interests have focused on two RAS regions, switch II pocket and the junction area of the switch I/II pocket, both of which are potential druggable pockets.^18^ The former was found to be an important region for covalent binding of drugs during the development of KRAS^G12C^ inhibitors, which could lock KRAS^G12C^ in an inactive form after binding to drugs,^111^ also named KRAS‐off inhibitors.^15^, ^86^, ^112^ The latter, as a conserved structure shared by all three RAS paralogs, has more potential as a pan‐RAS inhibitor targeting region, but how to improve the affinity of the drug for mutant RAS and make it much higher than for the wild‐type RAS is a thorny challenge.^113^ More recently, “KRAS‐on” inhibitors that target active KRAS–GTP have also been developed to block interactions of “on‐state” RAS with downstream effectors. In this strategy, cyclophilin‐A, as an intracellular chaperone protein of drug, binds to S‐IIP of RAS–GTP to form a tri‐complex of KRAS, cyclophilin‐A, and drugs, such as RMC‐6291.^114^ The tri‐complex leads to the covalent RAS cross‐linkage, subsequently blocking downstream effector binding to KRAS. Similarly, in the presence of the compound RM‐018, chaperone proteins block downstream signal transduction by forming tri‐complexes to block binding of RAS with RAF.^115^ This strategy provides more flexibility for investigating ways to specifically target RAS mutations.

In addition, recent studies have revealed that there are missense mutations at the same site or certain differences among mutations at different RAS sites, which may have implications for the development of drugs targeting specific RAS mutations. Unfortunately, there are few studies comparing the differences between paralogs at the same point. For example, a report shows significant phenotypic differences as well as divergence in signaling pathways of KRAS^G12D^ and NRAS^G12D^ in CRC.^116^

### 5.2 Specific inhibitors targeting mutated RAS

In addition to the non‐specific mutation inhibitors, the recent success in developing KRAS^G12C^ inhibitors has revived interest in developing KRAS inhibitors that either directly target KRAS mutations or target key steps required for KRAS activation. The research progress for specific targeted inhibitors of KRAS^G12x^ mutations (such as G12C) is still ahead of other mutant KRAS. Regrettably, efficacious targeted inhibitors of G13D and Q61R have not been found, nor have specific inhibitors of oncogenic NRAS and HRAS mutants.

#### 5.2.1 Drugs targeting KRAS^G12C^

##### 5.2.1.1 Introduction of KRAS^G12C^ inhibitors

KRAS^G12C^ with a high incidence in cancers such as non‐small cell carcinoma is a common type of KRAS mutation.^117^ Recently, researchers have found that there is a new pocket adjacent to the mutated cysteine residue (cys12) on the switch II region of the GDP‐binding KRAS^G12C^ protein. The developed small‐molecule targeted drugs could bind to the allosteric pocket by forming irreversible covalent compounds with cys12. Moreover, other inhibitors preferentially bind to the KRAS^G12C^–GDP and block SOS‐mediated nucleotide exchange and thereby inhibit the hyperactivation signals of RAS.^86^ The successes in clinical trials of these inhibitors make a breakthrough in the development of KRAS^G12C^‐specific compounds with anticancer activity in vivo for targeting the SII pocket. The information on the structure, covalent bond, and medication guidance of KRAS^G12C^ inhibitors is summarized in detail in several reviews. Dunnett‐Kane et al.^118^ provide a detailed description of the therapeutic activity of several registered KRAS^G12C^‐specific inhibitors and summarizes their clinical trial stages and trial designs. In addition, interestingly, in cancer cells that are insensitive or resistant to a covalent KRAS^G12C^ inhibitor ARS1620, Zhang et al.^119^ reported that ARS1620‐modified peptides in major histocompatibility complex I (MHC‐I) complex could serve as neoantigens. Subsequently, these neoantigens are recognized by recombinant antibody P1A4. The P1A4 induces cytolytic T‐cell response, so ARS1620‐resistant KRAS G12C mutant cells are killed in vitro. The strategy provides inspiration for reducing clinical resistance of single G12C inhibitors.

##### Therapeutic effect of RAS G12C inhibitors

Since the successful development of G12C mutation inhibitors was reported, various G12C inhibitors have been rapidly put into clinical trials. Among G12C inhibitors, sotorasib (AMG510) and adagrasib (MRTX849) are representative agents that have been shown to have direct therapeutic effects in various cancers with G12C mutations. Although targeted inhibitors of KRAS^G12D^ have also been developed, there are no clinical trial data. Therefore, this section will focus on the clinical progress of G12C inhibitors.

On the one hand, sotorasib was approved by the Food and Drug Administration as a second‐line treatment for locally advanced or metastatic non‐small cell lung cancer (NSCLC) containing the KRAS^G12C^ mutation.^120^ In the CodeBreaK100 phase I trial, 960 mg was identified as the recommended phase II dose by estimating the response of approximately 30 patients in the dose‐escalation cohort.^121^ After 11.7 months as the median follow‐up time, 32.2% of the patients in the subgroup with NSCLC had a confirmed response with a disease control rate of 88.1%. The median progression‐free survival (mPFS) for patients with NSCLC was 6.3 months. In the phase II portion of CodeBreaK100, among the 126 enrolled patients, 37.1% of the patients had a confirmed response with a disease control rate of 80.6% with a median follow‐up of 15.3 months. The mPFS was 6.8 months and the median overall survival (mOS) was 12.5 months. The rate of treatment‐related adverse events was 69.8%, including 19.8% probability of occurrence of grade 3 event and 0.8% probability of occurrence of grade 4 event.^112^

Also, sotorasib has shown monotherapy clinical activity in KRAS^G12C^‐mutated CRC in the CodeBreaK100 phase I trial, although the treatment is less effective. With a median follow‐up of 12.8 months, the response rate among patients was 7.1% with a disease control rate of 66.7%.^121^

On the other hand, the phase I/II KRYSTAL‐1 trial explored the therapeutic effect of adagrasib monotherapy.^122^ A dose of 600 mg twice daily was identified as the recommended phase II dose by estimating the response of 25 patients in the dose‐escalation cohort. Among 15 evaluable patients with KRAS^G12C^ NSCLC treated with adagrasib 600 mg bid, 53.3% of the patients had a confirmed response, with a median duration of response of 16.4 months. The mPFS was 11.1 months and the 12‐month survival rate was 66.7%.

Adagrasib is also being studied in CRC with KRAS^G12C^.^123^ With a median follow‐up of 12.8 months, the response rate among patients was 22%, with a disease control rate of 87%, and the mPFS was 5.6 months.

##### Resistance mechanism of G12C inhibitor monotherapy

In the clinical application of G12C inhibitors, it has been observed that there are differences in drug‐resistance involved pathways in different cancer types. In NSCLC, upregulation of MEK and ERK is found to develop for resistance of sotorasib. Differently, in CRC, the phosphorylation of EGFR leads to the resistance to G12C inhibitors.^94^, ^124^, ^125^ This suggests that for CRC carrying G12C, the combination of G12C inhibitors and EGFR inhibitors may be clinically more effective than G12C inhibitor monotherapy.

#### 5.2.2 Drugs targeting KRAS^G12D^

There are several studies on specific inhibitors targeting KRAS^G12D^. KRAS^G12D^ commonly occurs in digestive system cancers, especially pancreatic cancer and CRC. The lack of a highly reactive residue, such as the cysteine residue at position 12, renders targeted KRAS^G12D^ drug design in a distinct approach. Vatansever et al.^58^ performed computational simulations on the kinetic data of KRAS^G12D^ to explore the changes in the domain of mutated RAS, aiming to lay a foundation for subsequent targeted drug research. Similarly, through computational analysis of protein structures, Feng et al.^126^ found P110, the connection site adjacent to proline 110, and a small molecule named KAL‐21404358 that binds to this site (*K*_D_ = 100 μM). In addition, the compound 3144 developed by Stockwell et al.^127^ could target KRAS^G12D^ with micromolar affinity. Interestingly, when screening small‐molecule compounds targeting KRAS switch I/II pocket, Kessler et al.^113^ reported that a compound named BI‐2852, in contrast to covalent inhibitors, could bind to both the active and inactive forms of KRAS with nanomolar affinity. Recently, a specific inhibitor targeting KRAS^G12D^, named MRTX1133, has been shown to bind KRAS^G12D^ in a non‐covalent binding form (*K*_D_ = ∼0.2 pM). And the selectivity of MRTX1133 binding to KRAS^G12D^ is 700‐fold higher than that of binding to KRAS^WT^.^128^ In addition to small‐molecule compounds, there has been some progress in the development of peptides as another form of targeted drugs. For example, KRpep‐2d and its derived peptide KS‐58 are developed to selectively bind to the KRAS^G12D^–GDP with sub‐nanomolar *K*_D_ values and inhibit nucleotide exchange of KRAS^G12D^.^129^ Table 3 summarizes the characteristics of mutated KRAS inhibitors (except G12C).

**TABLE 3:** Features of non‐G12C KRAS inhibitors.

Targeted mutation	Name	Structure	Reactive functional group	Targeted position in RAS	*K*_D_	Reference
G12D	KAL‐21404358	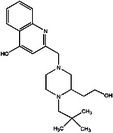	Quinolinol and piperazinyl group	Proline 110	100 μM	^126^
	3144	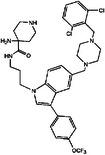	N/A	S39, D38, E37, and I36	∼20 μM	^130^
	BI‐2852	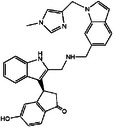	Isoindolinone	SI/II pocket	750 nM	^113^
	MRTX1133	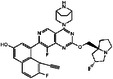	N/A	S‐II pocket	∼0.2 pM	^128^
	KS‐58	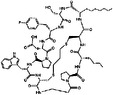	Amide bond cyclization and main chain cyclization	S‐II pocket	100 nM	^129^
G12V	H‐REV107	N/A	L65, Y66, D67, G70, D72, K73, and Y74	SI/II pocket and P‐loop	30 μM	^131^
	TKR15	N/A	Thiourea and benzotrifluoride group	N/A	N/A	^132^
G12S	G12Si‐5	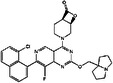	β‐Lactone	S‐II pocket (Ser12)	26 μM	^133^
G12R	N/A	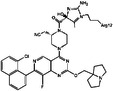	α,β‐Diketoamide	S‐II pocket (ε‐N and η‐N of R12)	N/A	^134^

The structure of the chemical formula in Table 3 is drawn with ChemDraw.

#### 5.2.3 Drugs targeting KRAS^G12V^

Currently, the clinical trials for specific inhibitors of KRAS^G12V^ are rare. Han et al.^131^ developed a derived peptide based on H‐REV107 in vivo that could interact with KRAS^G12V^–GDP to form a stable complex, thereby blocking the activating function of KRAS and inhibiting phosphorylation level of MEK/ERK.

In addition, there is a report finding that tyrosine kinase receptor 15 (TKR15), a thiourea derivative, is able to inhibit cell proliferation and induce apoptosis in A549 cells by targeting KRAS^G12V^.^132^

#### 5.2.4 Drugs targeting other KRAS mutations

In the development of KRAS^G12R^ inhibitors, Zhang et al.^134^ screened α,β‐diketoamide from common electrophilic ligands based on the nucleophilicity of arginine residue in KRAS^G12R^. The research reveals that the ligand is able to selectively bind to KRAS^G12R^ in a covalent form, providing reference for the development of inhibitors targeting KRAS^G12R^.

In the development of KRAS^G12S^ inhibitors, Zhang et al.^133^ also chose the nucleophilicity of serine residue as the weakness of KRAS^G12S^. The potential of β‐lactone derivatives as ligands for KRAS^G12S^ is revealed, and Ser12 in SIIP is acylated by the carbonyl group of β‐lactone during ligand formation of a covalent complex with KRAS^G12S^.

## 6 INDIRECT MUT‐RAS INHIBITION: INTERVENTION IN DOWNSTREAM PATHWAYS

**FIGURE 3:**
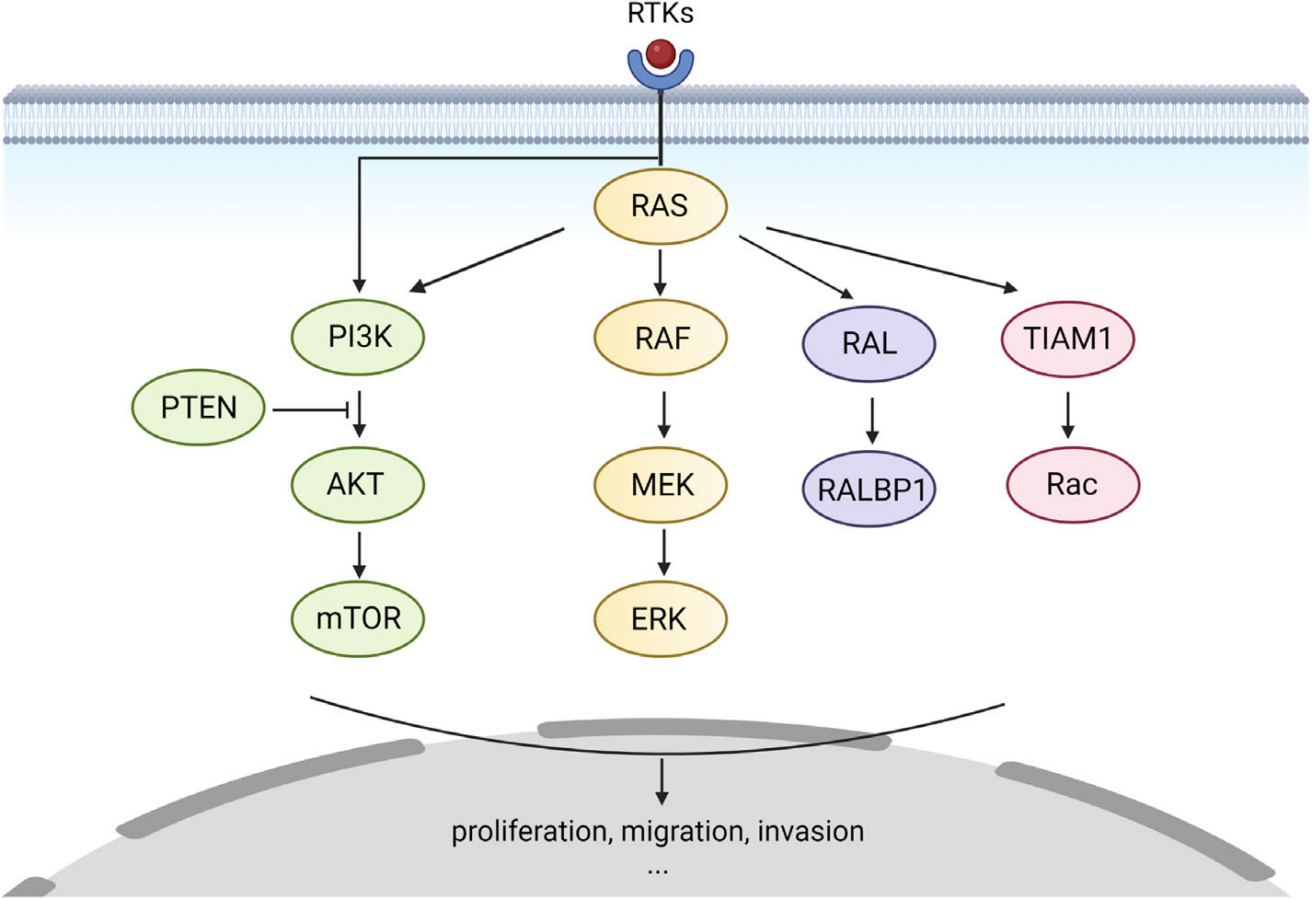
Downstream signaling pathway of KRAS. After receiving the signal of epidermal growth factor, receptor tyrosine kinases (RTKs) such as EGFR will recruit RAS, targeting the membrane and activating it. Therefore, phosphorylation activation signals are passed in the downstream cascades, which contain the RAF–MEK–ERK, PI3K–protein kinase B (AKT), RAL, and TIAM1 pathways. These cascades regulate cell proliferation, migration, and invasion. The figure was made using Biorender.

RAS, as an important component in regulating cell growth and proliferation, is involved in a large network of related cascaded signaling pathways. In the development of anticancer drugs, it is essential to understand the signaling pathways associated with RAS.^115^, ^135^ Overactivation of the RAF/MEK/ERK and phosphatidylinositol‐3‐kinase (PI3K)/protein kinase B (AKT)/mechanistic target of rapamycin (mTOR) pathways enhances growth, survival, and metabolism of cancer cells. Thus, the signaling pathways have been identified as promising therapeutic targets for cancer therapy.^136^ This section focuses on the RAS downstream pathways shown in Figure 3 that are closely associated with the development of cancers with RAS mutation. The impact of pathways for specific cancer types is summarized as well.

### 6.1 Intervention in RAF–MEK–ERK (MAPK) pathway

#### 6.1.1 Components of MAPK pathway

The RAS/mitogen‐activated protein kinase (MAPK) pathway, as a classical downstream signaling pathway of RAS, is closely related to variety of cancers. MAPK is a phosphorylated activation effector cascade composed of RAF, MEK, and ERK protein kinases, regulating cell growth, survival, proliferation, and differentiation.^137^, ^138^, ^139^, ^140^ RAF is the direct effector of activated RAS as the beginning of cascade signals. And there are three proteins in RAF family, ARAF, BRAF, and CRAF. In cascade signaling, RAF is recruited through its RBD domain selectively binding to the active GTP–RAS on the plasma membrane.^141^, ^142^ Subsequently, the cysteine‐rich domain (CRD) domain of RAF selectively binds to the farnesyl groups of modified RAS, which further promotes the recruitment of RAF to cell membrane.^143^, ^144^ On the basis of RBD binding to RAS, CRD also interacts with phosphatidyl serine in the plasma membrane.^145^ During the recruitment, affected by the increase in local concentration and conformational change of RAF,^146^, ^147^ its homologous dimerization and heterodimerization with any other RAF family member will be induced, which also promotes the activating phosphorylation of RAF.^148^ After activating the downstream MEK/ERK, RAF kinase reverts to an inactive conformation due to complex regulation, such as dephosphorylation by kinases,^149^ autoinhibition by amino‐terminal domain, and negative feedback regulation of ERK.^150^, ^151^, ^152^ However, once RAF is mutated, the above complex regulation of RAF activation will be disrupted. Oncogenic BRAF mutations are common in cancer.^153^ One class of BRAF mutants, BRAF‐V600 mutants, will lose its autoinhibitory activity and also be activated continuously without relying on RAS or dimerizing. Another class of mutants will similarly be RAS independently activated, which still relies on dimerization for complete activation. The third mutants, stimulating BRAF^WT^ to form heterodimers, are kinase‐impaired proteins and need RAS‐binding activation and dimerization.^154^, ^155^, ^156^, ^157^

Similar to RAF, a region of the N‐terminal lobe (negative regulatory region) of MEK kinase also exists for autoinhibition.^158^ In the process of MEK activation by RAF phosphorylation, kinases such as kinase suppressor of RAS (KSR) act as scaffold proteins to assist the assembly of RAF–MEK–ERK complex and promote RAF to activate MEK.^159^, ^160^ After the complex forms, the serine residues of MEK (S218 and S222 of MEK1, S222 and S226 of MEK2) are diphosphorylated rapidly for MEK activation.^161^, ^162^ Subsequently, tyrosine and threonine residues of downstream ERK (T202/Y204 of ERK1 and T183/Y185 of ERK2) are dephosphorylated.^163^ Unlike RAF kinase, MEK/ERK mutations are rare in human tumors.^164^, ^165^

#### 6.1.2 The role of MAPK in cancer

In driving the development of RAS‐mutated cancers, the activity of MAPK kinases varies among different tumors. Furthermore, RAF subtypes have different influences on RAS‐driven tumors. For example, BRAF is dispensable for NSCLC with KRAS^G12V^ or KRAS^G12D^, caused by the other RAF kinases presenting a compensatory effect without increased expression. On the contrary, CRAF is essential to mediate oncogenic signaling in the same cancer.^166^, ^167^ However, CRAF deficiency does not affect tumor development of pancreatic ductal carcinoma (PDAC) with KRAS^G12D^ or KRAS^G12V^, while concomitant ablation of EGFR and CRAF completely prevent PDAC development driven by KRAS^G12V^/Trp53 mutation.^168^, ^169^ However, the selection of CRAF as a therapeutic target tends to rely on ablation rather than inhibition of kinase activity. On the one hand, clinical inhibition of CRAF kinase activity requires prevention of inhibition of BRAF kinase activity, thereby avoiding increased toxicity.^170^ On the other hand, CRAF ablation regulates apoptotic pathway‐related proteins independent of kinase activity or MAPK pathways, which may support CRAF ablation to inhibit tumor development.^171^ In addition to apoptosis‐related proteins, CRAF regulates many proteins in a kinase‐independent manner. For example, CRAF inhibits the kinase activity of ROKα, and the ablation of RAF1 induces the regression of squamous tumors via ROKα‐mediated cellular differentiation.^172^

Unlike RAF, systemic ablation of MEK or ERK will induce the unacceptable toxicities in adult mice, although preventing tumor development.^170^ Interestingly, in the CRC model with KRAS^G12V^, heterogeneity in intracellular ERK phosphorylation has been observed. ERK levels are generally higher in cancer cells adjacent to stromal cells at the invasive front and lower in more central areas of cancer specimens.^173^

#### 6.1.3 Clinical effect of MAPK inhibitors

Although the MAPK is a linear cascade, the regulation of each element involves multiple kinases. Therefore, in the treatment with inhibitors, the regulation of feedback pathways in vivo after drug administration should be considered to avoid drug resistance.

Significant efforts have been made to develop inhibitors targeting the MAPK pathway, especially BRAF and MEK inhibitors. However, only the BRAF inhibitors vemurafenib and dabrafenib and the MEK inhibitors trametinib and cobimetinib have been approved only for BRAF‐V600E/K metastatic melanomas.^174^ In KRAS‐mutated tumors, BRAF inhibitors promote heterodimerization of BRAF and CRAF, thereby activating the MAPK pathway and helping secondary tumor development.^175^, ^176^, ^177^ Recently, some novel pan‐RAF inhibitors have been investigated to solve the problem of promoting dimerization. LY3009120 inhibits MEK1/2 phosphorylation by inhibiting kinase activity in BRAF–CRAF heterodimers and retards the development of tumors carrying KRAS mutations while inducing a more significant dimerization.^178^ However, when used as a single agent, the required dose of RAF inhibitor being effective in KRAS‐mutated models is significantly higher than that in the BRAF‐V600E model. Therefore, it is necessary to continue upgrading effective drugs or treat with a combination of drugs.

Similar to RAF inhibitors, cobimetinib also fails to inhibit KRAS mutation‐carrying tumors due to RAF feedback activation of MEK phosphorylation.^179^ In contrast, trametinib appears to inhibit the proliferation of KRAS mutant A549 cells effectively by impairing the RAF–MEK interaction.^180^ However, its resistance still exists in KRAS mutant NSCLC, which is caused by compensatory activation of FGFR1.^181^ As a result, MEK inhibitors are increasingly used in combination with other inhibitors to avoid drug resistance due to the feedback regulatory mechanism induced by their use alone.

As for ERK inhibitors, the drug resistance tends to develop after monotherapy with ERK1/2 inhibitor over a period of time.^182^, ^183^ One of the mechanisms is the compensatory effect of ERK5 in place of the inactivated ERK1/2.^184^ In response, inhibitors against ERK5 have been developed. On the other hand, ERK inhibition blocks negative feedback of ERK and induces feedforward activation of upstream RTK, thereby inducing activation of alternative pathways such as the PI3K/AKT pathway to maintain tumor cell survival. Therefore, inhibitors targeting both ERK1/2 and other kinases are also developed for treatment.^185^ For example, Gao et al.^186^ found that an indole‐substituted pyrimidine derivative inhibits the activities of AKT and ERK1/2, thereby inhibiting tumor growth and extending the survival time of tumor‐bearing mice.

As a significant downstream signaling pathway of RAS, MAPK has become a popular therapeutic target. However, the efficacy of MAPK inhibitors in monotherapy of tumors with RAS mutation is not ideal. According to the above, one of the reasons is that the MAPK pathway is abnormally activated after the suppression of components. The mechanisms of reduced effectiveness or resistance of inhibitors vary according to the target and how the inhibitor works. On the other hand, the toxicity caused by inhibitors is also worthy of attention.^187^ Therefore, rather than monotherapy, combinations of drugs targeting the MAPK pathway have been studied more frequently in the clinical treatment of cancers carrying RAS mutations.

### 6.2 Intervention in PI3K/PTEN/AKT/mTOR pathway

#### 6.2.1 Components of PI3K pathway

Similar to MAPK pathway, PI3K pathway is an effector cascade dependent on phosphorylated activation. PI3K is divided into three classes: I, II, and III. Class I PI3Ks are expressed in various cell types and are related to development of cancers.^188^, ^189^, ^190^, ^191^, ^192^ Therefore, class I PI3Ks are selected to be mainly discussed in this section. Class I PI3Ks are heterodimeric proteins that are grouped into two subtypes: IA and IB. PI3K IA proteins are composed of a regulatory subunit (p85α, p85β, p50α, p55α, p55γ) and a 110‐kDa catalytic subunit (p110α, p110β, p110δ). PI3K IAs act as downstream kinases of TKRs and G protein‐coupled receptors (GPCRs). PI3K IBs have a p110γ catalytic subunit binding p101 or p87 as regulatory subunit. Different from PI3K IAs, PI3K IBs are activated by GPCRs.^193^, ^194^, ^195^ Class I PI3Ks are activated through different upstream mechanisms, which mainly contain: (1) the regulatory subunit p85 binding to phospho‐YXXM motifs (X indicates any amino acid) of the RTK, thereby triggering activation of the catalytic subunit p110^196^; (2) growth factor receptor‐bound protein 2 (GRB2) binding to phospho‐YXN motifs of the RTK in advance and to the scaffolding protein GAB, which in turn can bind to p85^197^; and (3) GRB2 binding to RTK and subsequently activating SOS, RAS in turn, and finally activating p110 independently of p85.^198^

After being activated, catalytic subunit of PI3Ks transfers the phosphate group to PIP2 to produce PIP3. In the course, phosphatase and tensin homolog deleted on chromosome 10 (PTEN), a negative regulator, acts as a phosphatase to dephosphorylate PIP3 and convert it back to PIP2. Back to the downstream signaling pathway, PIP3 recognizes downstream proteins with a pleckstrin homology domain, such as PDK1 and AKT, and recruits them to the cell membrane.^199^, ^200^ In the cascade, AKT is partly activated by phosphorylation of PDK1 at T308. Subsequently, mTORC2 will fully activate AKT through phosphorylation of AKT at S473.^201^, ^202^ Then, many downstream effectors, which are involved in protein synthesis, cellular proliferation, apoptosis, and cell survival, such as mTOR, are phosphorylated by AKT.^203^, ^204^, ^205^, ^206^, ^207^, ^208^

#### 6.2.2 The role of PI3K pathway in cancer

PI3K pathway activated by RAS is essential for lung carcinogenesis driven by KRAS^G12D^.^209^ However, the established tumors are less dependent on PI3K signaling and PI3K/mTOR inhibition only leads to partial tumor regression.^209^, ^210^ Similarly, p110α of PI3K inactivation dose dependently can prevent mouse lethality and the occurrence of cancers induced by KRAS^G12D^. p110α activity is also required for in vivo superactivation of KRAS^G12D^ and other signaling pathways.^168^, ^211^

#### 6.2.3 Clinical effect of PI3K pathway inhibitors

PI3K pathway has also been selected as a target for cancer therapy due to the discovery of overactivation of PI3K in a variety of cancers and its significance for proliferation and survival of cancer cells. However, in the course of treatment, problems such as abnormal activation of feedback, compensation activation, drug resistance, and toxicity of PI3K pathway inhibitors are found.^212^, ^213^, ^214^

### 6.3 Intervention in other pathways

As a downstream effector of RAS, RAL GTPase mediates various cellular activities to regulate tumor invasion, proliferation, and resistance to cell death, which are executed by the RAL effectors, including RALBP1, Sec5, Exo8426, Filamin, PLD1, and ZONAB.^215^, ^216^, ^217^, ^218^, ^219^, ^220^, ^221^ RAL is grouped into two subtypes: RALA and RALB. The former plays a major role in cancer development and metastasis. In NSCLC, growth of cancer cells carrying KRAS^G12C^ is more sensitive to RAL depletion.^222^ As for in pancreatic cancers, RALA is essential for tumor growth, and RALA and RALB are both required for tumor invasion.^223^, ^224^

In addition of RAL, TIAM1 can bind to RAS through its RAS‐binding domain, causing synergistic formation of Rac‐GTP in a PI3K‐independent manner, thereby activating Rac to induce activation of the NF‐kappa B transcription factor and promotion of cancer cell survival.^225^ However, in epithelial MDCK cells, Tiam1–Rac and RAS signaling seemingly oppose each other, since RAS^G12V^‐induced epithelial–mesenchymal transition is negatively affected by Tiam1–Rac signaling.^226^, ^227^

## 7 COMBINATION STRATEGIES ADAPTED TO SPECIFIC MUTATIONS IN DIFFERENT CANCERS

After a long period of struggle in developing RAS‐targeted drugs, researchers have turned their attention to the RAS downstream signaling pathway inhibitors, in order to indirectly inhibit RAS activity. RAS would be considered as an important component of the signaling pathway that regulates cell growth and proliferation. The upstream activation network of RAS is quite complex, and the downstream signaling pathway network is highly complicated as well, both of which lead to intricate cross‐talk between RAS‐related pathways. Therefore, protein targeting in a pathway in the RAS‐related signaling network often fails to achieve the anticipated therapeutic purpose. Indeed, it is found in clinical treatment that specific targeted therapy or specific drug combination has an unexpected effect on the cancers caused by different RAS mutations. In this section, we mainly list the treatment of several cancers commonly carrying KRAS mutations, such as pancreatic cancer, NSCLC, and CRC. Table 4 summarizes the progress of clinical trials on direct KRAS inhibitors for cancers carrying known KRAS mutations with published results in the last 5 years. In addition, as a supplement, Table 5 shows ongoing clinical trials for cancers with KRAS mutations (except for KRAS^G12C^).

**TABLE 4:** Efficacy of clinical trials adapted to cancers with mutated KRAS in last 5 years.

Cancer	Mutation	Drug	Study phase	Response rate (*N*)	DR (months)	PFS (months)	OS (months)	Reference
PC	G12C	Sotorasib	I/II	21% (38)	N/A	4.0	6.9	^228^
	G12R	Selumetinib sulfate	II	0% (8)	N/A	3.0	N/A	NCT03040986
CRC	G12C	Sotorasib	I	7.1% (42)	N/A	4.0	N/A	^121^
		Adagrasib	I/II	50% (2)	4.2	N/A	N/A	^122^
		Adagrasib	I/II	19% (43)	4.3	5.6	N/A	^229^
		Adagrasib with cetuximab		46% (28)	7.6	6.9		
		JNJ‐74699157 (ARS‐3248)	I	25% (4)	N/A (safety deficiency)			^230^
NSCLC	G12C	AMG510 (sotorasib)	I	90% (10)	1.9–5.9	N/A	N/A	^231^
		Sotorasib	I	32.2% (59)	10.9	6.3	N/A	^121^
			II	37.1% (126)	11.1	6.8	12.5	^112^
		Docetaxel	III	13.2% (129)	6.8	4.5	11.3	^232^
		Sotorasib		28.1% (158)	8.6	5.6	10.6	
		Adagrasib	I/IIb	53.3% (15)	16.4	11.1	N/A	^122^
		Adagrasib	II	42.9% (116)	8.5	6.5	12.6	^233^
		JNJ‐74699157 (ARS‐3248)	I	60% (5)	N/A (safety deficiency)			^230^
	G12D	Bortezomib with acyclovir	II	41.2% (17)	N/A	1	13	NCT01833143

Abbreviations: CRC, colorectal cancer; DR, duration of response; NSCLC, non‐small cell lung cancer; OS, overall survival; PC, pancreatic adenocarcinoma; PFS, progression‐free survival.

**TABLE 5:** Ongoing clinical trials for cancers with KRAS mutations (except for G12C).

Trial ID	Tested interventions	Treatment setting	Phase	Status
NCT05533463	HRS‐4642	Advanced KRAS^G12D^ mutant solid tumors	I	Recruiting
NCT05737706	MRTX1133	KRAS^G12D^ mutant solid tumors	I/II	Recruiting
NCT04853017	ELI‐002 2P	PDAC with KRAS^G12D^ and KRAS^G12R^	I	Recruiting
NCT05631899	KRAS‐EphA‐2‐CAR‐DC abraxane cyclophosphamide PD‐1 antibody	Solid tumors with KRAS^G12V^	I	Recruiting
NCT04146298	Cyclophosphamide fludarabine PD‐1 antibody Biological: G12V‐specific TCR transduced autologous T cells	Advanced PDAC with KRAS^G12V^	I/II	Recruiting
NCT04620330	Avutometinib (VS‐6766) or/and defactinib	NSCLC with KRAS^G12V^	II	Recruiting

*Note*: The data originated from https://clinicaltrials.gov.

Abbreviations: NSCLC, non‐small cell lung cancer; PD‐1, programmed death‐1; PDAC, pancreatic ductal carcinoma; TCR, T cell receptor.

### 7.1 Therapy of KRAS‐mutated pancreatic cancer

#### 7.1.1 Therapy of pancreatic adenocarcinoma with KRAS^G12D^

First of all, considering the successful development of inhibitors targeting KRAS^G12D^ mutation, the therapeutic effect of KRAS^G12D^ inhibitors on pancreatic adenocarcinoma (PC) is preferentially discussed. As a non‐covalent KRAS^G12D^ inhibitor, MRTX1133 inhibits phosphorylation levels of ERK1/2 and cell viability in KRAS^G12D^ mutant cell lines (IC_50_ = ∼5 nM). Based on this, MRTX1133 exhibited obvious tumor regression (≥30%) in KRAS^G12D^ mutant PDAC models.^128^ In addition to the efficacy, it is also found that combination of MRTX1133 and inhibition of EGFR or PI3Kα, which are members of potential feedback or bypass pathways, improves the antitumor activity in PDAC. As another means of targeting G12D mutations, combination of siG12D‐LODER with gemcitabine and FOLFIRINOX exhibits good tolerance and certain inhibition of disease progression in patients with locally advanced pancreatic cancer with KRAS^G12D^.^234^ Similarly, silence of KRAS^G12D^ by CRISPR‐CasRx appears to suppress the tumor growth, enhance the sensitivity of gemcitabine in PDAC, increase the survival of mice, and show obvious toxicity.^235^

From the downstream pathway inhibitor perspective, trametinib (MEKi) and ruxolitinib (STAT3i) help nivolumab (programmed death‐1 [PD‐1] inhibitor) improve antitumor activity and survival in mice carrying *Ptf1a^Cre/+^*, *LSL‐Kras^G12D/+^*, and *Tgfbr2^flox/flox^* tumors. More importantly, the combination strategy results in a clinical benefit for a patient undergoing chemotherapy‐refractory metastatic PDAC.^236^

Despite the above‐mentioned therapies, immunotherapy is also receiving much attention. A study revealed that inhibition of interleukin‐6 enhances the antitumor effect of anti‐programmed death‐1‐ligand 1 (PD‐L1) checkpoint inhibitor therapy. The treatment combination delayed tumor progression (*p* < 0.001) and increased OS in engineered PDAC model with KRAS^G12D^ (*p* = 0.0012).^237^

Recently, antidiabetic drug metformin was excavated to have antitumor activity in PDAC induced by KRAS^G12D^. In LSL‐*Kras^G12D/+^*, *Trp53^fl/+^*, and *Pdx1‐Cre* (KPC) mouse model, metformin has ability of blocking tumor growth, inhibiting the incidence of abdominal invasion, and increasing the OS.^238^ In addition, Si‐HSF1 was found to increase chemosensitivity to gemcitabine in vivo.^239^

#### 7.1.2 Therapy of PC with KRAS^G12V^

In terms of targeting G12V drugs, Ghufran et al.^240^ have designed peptides inhibiting KRAS^G12V^, which are speculated to have the ability to inhibit G12V activity and reduce the progression of cancer. In spite of targeting RAS itself, a dual FT and geranylgeranyltransferase‐1 inhibitor named FGTI‐2734 can inhibit the growth of xenografts derived from four patients with pancreatic cancer with mutant KRAS (two G12D and two G12V) tumors.^241^

In the aspect of immunotherapy, it has been reported that the combination of anlotinib and PD‐1 inhibitor and chemotherapy exhibits a long‐term partial response and good tolerance in a young patient suffering from PDAC with liver metastasis.^242^

In other ways, sequential administration of cell‐cycle kinases CDK4 and CDK6 inhibitors after taxane treatment reduced cell proliferation in PDAC mouse model with both KRAS^G12V^ and Cdkn2a‐null mutations.^243^ Diego‐González et al.^244^ found that lipoplexes of si‐FOSL‐1 and si‐YAP reduce the tumor growth in mice carrying tumors induced by pancreatic tumoral cell lines (KRAS^G12V^ and p53 knockout) through peri‐tumoral injection.

#### 7.1.3 Therapy of PC with KRAS^G12R^

Pancreatic cancers with KRAS^G12R^ are studied less than the former. Recently, it was found that KRAS^G12R^ is defective for interaction with p110α of PI3K, while PI3Kγ will support macropinocytosis in KRAS^G12R^ mutant PDAC in a compensatory way.^245^, ^246^ As for the therapy taken in PC with KRAS^G12R^, the administration of selumetinib as MEK1/2 inhibitor appears to have little effect.^247^ This suggests that single‐agent MEK inhibition is unable to meet the therapeutic needs of patients suffering from pancreatic cancer with G12R. And the combination of cobimetinib (MEK1/2 inhibitor) and gemcitabine improves PFS and OS after treatment in patients with KRAS^G12R^ compared with pancreatic cancer patients with KRAS^G12D/G12V^.^95^

### 7.2 Therapy of KRAS‐mutated CRC

#### 7.2.1 Therapy of CRC with KRAS^G12D^

Different from the therapeutic effect in pancreatic cancer, MRTX1133 has a lower inhibitory effect in CRC carrying KRAS^G12D^.^128^ In contrast to PDAC, KRAS mutations are usually not considered an initial driver in CRC, which may be one of the reasons for the limited effect of KRAS^G12D^ inhibitors in CRC patients.^248^ Similarly, the G12D‐targeting pathway is peptide KRpep‐2d. Similarly, peptide KRpep‐2d, a G12D‐targeting inhibitor, had no significant antitumor effect on the PDX model, while oxaliplatin showed a significant inhibitory effect.^249^ The failure of KRpep‐2d is suspected to be related to bioavailability and stability.^250^ Additionally, the combination of binimetinib, hydroxychloroquine, and bevacizumab makes a 17% reduction in lung metastasis size in FOLFOX‐resistant patients after 6 weeks treatment with this combination with FOLFOX.^251^

#### 7.2.2 Therapy of CRC with KRAS^G12V^

The combination of low‑dose trametinib (10 nM) and ABT263 (Bcl‑xL inhibitor) was found to inhibit tumor growth against a KRAS^G12V^ xenograft.^252^ In spite of targeting MEK and Bcl‑xL, miR‐4689 is also found to exhibit a potent growth inhibitory and proapoptotic effect by directly targeting KRAS^G12V^ and AKT1.^253^

#### 7.2.3 Therapy of CRC with KRAS^G13D^

Phase III clinical trial evidence suggests that CRCs with the KRAS^G13D^ may benefit from EGFR inhibitors, such as cetuximab, in contrast to the other most common KRAS mutations.^254^ Therefore, the therapy of CRC with KRAS^G13D^ mainly revolves around cetuximab.^255^, ^256^, ^257^ Chu et al.^258^ revealed that 4‐AAQB could resensitize KRAS mutant cells to cetuximab before cells were treated with cetuximab. In another study, the combination of first‐line chemotherapy drugs such as FOLFOX and cetuximab improved OS and PFS after treatment in chemotherapy‐refractory CRC patients with KRAS^G13D^, while no response was observed in CRC cell lines with KRAS^G12X/Q61X^ mutations or KRAS^WT^ CRC cell lines with BRAF mutations or no expression of PTEN or EGFR proteins.^259^, ^260^

Resistance of EGFR inhibitors in CRC with KRAS^G13D^ tends to depend on tumor suppressor NF1. NF1 can convert GTP–KRAS^G13D^ to GDP–KRAS^G13D^, and the resistance may be caused by impaired interaction between KRAS^G13D^ and NF1.^254^, ^261^

#### 7.2.4 Therapy of CRC with other KRAS alleles

The combination of irinotecan and cetuximab is administered in patients with KRAS^WT^ mCRC who responded to first‐line chemotherapy with cetuximab and developed a certain therapeutic effect after cetuximab in second‐ and third‐line treatment.^262^

The combination of KRAS^G12C^ inhibitor and EGFR inhibitor targets the rare CRC that carries the G12C mutation. There is a lower response rate to KRAS^G12C^ inhibitors alone in CRC patients than in NSCLC patients because of RTK–SHP2‐mediated adaptive RAS reactivation. The combination of cetuximab can enhance the efficacy of AMG510 in KRAS^G12C^‐mutated CRC PDX model.^125^, ^263^

### 7.3 Therapy of KRAS‐mutated NSCLC

#### 7.3.1 Therapy of NSCLC with KRAS^G12C^

Benefiting from the successful development of KRAS^G12C^ inhibitors, the current therapeutic strategy for NSCLC with G12C mutation is to combine KRAS^G12C^ inhibitors with other related pathway inhibitors, targeting immune checkpoints, EGFR, SHP2, SOS1, MEK, PI3K, mTOR, CDK4/6 or others, to improve the therapeutic effect.^264^, ^265^, ^266^, ^267^, ^268^, ^269^ Nevertheless, the problem of resistance to G12C inhibitors needs to be focused.

#### 7.3.2 Therapy of NSCLC with KRAS^G12V^

In the inhibition of RAS‐related pathway, it is showed that cotreatment with trametinib and osimertinib resensitizes the EGFR mutant NSCLC cell line with KRAS^G12V^ to osimertinib.^270^ A clinical study revealed that patients in the treatment of taxane cooperating with bevacizumab had higher objective response rate (ORR) than those treated with taxane alone.^271^ In another study, the combination of selumetinib and docetaxel improved PFS and ORR in patients with locally advanced or metastatic KRAS^G12V^ NSCLC (stage IIIB/IV), while no significant trend differences were observed due to the small statistical sample base.^272^

#### 7.3.3 Therapy of NSCLC with KRAS^G12D^

One patient with high tumor mutational burden and positive PD‐L1 expression with EGFR^L858R^ and KRAS^G12D^ mutations received therapy with a combination of bevacizumab, camrelizumab, and pemetrexed and achieved remission over 17 months.^273^, ^274^ In another clinical study, a series of therapy of panitumumab concomitant with radiation therapy and concurrent chemotherapy with paclitaxel and carboplatin was settled. During therapy, KRAS^G12D^ lung cancer clone of the patient with stage III NSCLC appeared to disappear.^275^

## 8 CONCLUSIONS AND PERSPECTIVE

Cancer therapies performed by targeting allelic RAS mutations in specific clinical contexts could be regarded as the milestone in personalized cancer treatment. After decades of exploration, there are advanced understandings of the structural differences, biochemical characteristics, and downstream signaling preferences of RAS mutations. These studies provide a solid foundation for designing effective targeted therapy for cancers harboring specific RAS mutations. Especially, successful development of KRAS^G12C^ inhibitors demonstrates the feasibility of developing specific therapeutic strategies for each RAS mutation. However, there are few studies on the structure and biochemical differences of other RAS mutants, such as KRAS^Q61L^, as well as HRAS and NRAS mutants. This may also be one of the reasons for the lack of effective inhibitors targeting other mutants other than KRAS^G12C^. In addition, there are currently few publicly recognized and effective treatment strategies for different cancer types with different RAS mutations at different stages. Hopefully, this review would provide some insights into individualized RAS inhibitor exploration and the refinement of drug combination for personalized cancer treatment strategy.

In the treatment of cancers induced by mutated RAS, it is necessary to identify the types of RAS mutations and the overactivated signaling pathways in specific cancer tissues. The experimental examples mentioned above have shown the differences in pathology of mut‐RAS‐induced cancer and related signaling pathways, which will lead to the effectiveness of the same treatment strategies. Table 6 summarizes the association of the development of various tumors with RAS mutations and downstream pathway proteins.

**TABLE 6:** Role of RAS pathways in related cancers.

Cancer	Mutation	Role of mutation in cancer	Essential pathway component	Role of component in cancer
PC	KRAS mutation	Inducing early PC^276^	–	–
		Promoting tumor metastasis and aggressiveness^277^, ^278^	RAL	Essential for tumor growth and invasion^223^, ^224^
		Maintaining tumor^279^	–	–
	KRAS^G12D/G12V^	–	CRAF	Dispensable for tumor development^168^, ^169^
	KRAS^G12C/G12D/Q61X^	Increased autophagic flux after suppression of KRAS^280^, ^281^	ERK	Increased autophagic flux after suppression of ERK^280^, ^281^
CRC	KRAS^G12X/Q61X^	KRAS mutations are usually not considered an initial driver^248^, ^254^	MAPK and PI3K/Akt	Low activation states in the primary tumors^282^
	KRAS^G13D^	CRC with G13D will response to EGFR inhibitors^254^	–	–
NSCLC	KRAS^G12C/G12V^	–	RAL	Increased activation^85^
		–	AKT	Decreased growth factor‐dependent AKT activation ^282^
	KRAS^G12D/G12V^		CRAF	Essential for tumor development^166^, ^167^
	KRAS^G12C^	–	RAL	Tumor inhibition after RAL depletion^222^
	KRAS^G12D^	–	PI3K	Increased activation^282^
		–	MEK	No obvious activation^282^
	KRAS^Q61H^	Increased dependence of tumor development on MAPK^283^	RAF/MEK	Increased activation and essential for tumor development^283^
Endometrial cancer	KRAS mutation	Inducing early EC^284^ and development	PI3K	Increased activation^285^
		Promoting type I EC aggressiveness^286^	MAPK PI3K	Increased FGFR‐dependent activation^287^
Neuroblastoma	NRAS^Q61V^	Promoting tumor development^288^	MAPK	Increased activation^288^
Breast cancer	HRAS^G12V^	Inducing proliferation signal^289^	PI3K	Increased activation^289^
	KRAS^G12V^	Maintaining mesenchymal characteristics and metastatic behavior^290^	mTORC1	Increased activation^291^

Abbreviations: AKT, protein kinase B; CRC, EC, endometrial cancer; colorectal cancer; MAPK, mitogen‐activated protein kinase; NSCLC, non‐small cell lung cancer; PC, pancreatic adenocarcinoma; PI3K, phosphatidylinositol‐3‐kinase.

In addition to the treatment options of targeting RAS and downstream pathway proteins, new treatments have also been developed in recent years. First, there are studies exploring new therapeutic strategies from the perspective of energy metabolism. In cancers caused by RAS overactivation, energy metabolism‐related features such as increased glycolysis rate^292^ and increased mitochondrial autophagy^293^ adapted to cancer cell growth would be detected. Therefore, there are studies attempting to inhibit the development of RAS‐related cancers by inhibiting the above pathways. Bryant et al.^280^ found the phenomenon of increased autophagy flux after inhibition of KRAS and MAPK pathways and demonstrated that autophagy inhibitor chloroquine and ERK inhibitor could synergically enhance the antitumor activity of KRAS‐driven PDAC. Therefore, the combination of MAPK inhibitor and autophagy inhibitor may be an effective treatment for PDAC. Secondly, the research on inhibiting RAS oligomerization is carried out. After RAS is localized in the membrane, oligomerization or dimerization of RAS occurs, which is necessary for effective RAS‐driven signaling.^294^ Based on the phenomenon, nano‐antibody NS1 was developed to disrupt HRAS–KRAS autocorrelation by directly binding to the α4–α5 interface, reducing downstream pathway activation and inhibiting cell proliferation while maintaining RAS localization and GTPase activity unaffected.^295^ In addition, there are other novel therapeutic methods, such as proteolysis‐targeting chimeras (PROTACs)^296^ and small‐molecule RNA interference technology,^234^ which also provide more selectivity for the treatment of RAS‐related cancers.

With the continuous development of mutant RAS inhibitors and other novel therapies, the drug combinations available for clinical use are more diverse. It is hoped that personalized cancer treatment research will make more targeted use of these drug combinations to continuously optimize the clinical therapeutic effect.

## AUTHOR CONTRIBUTIONS

C.L. wrote the paper. D.Y., X.L., Z.S., M.D., and Y.L. revised the manuscript. H.Y. and X.C. made the figures. All authors have read and approved the final manuscript.

## CONFLICT OF INTEREST STATEMENT

The authors declare they have no conflicts of interest.

## ETHICS STATEMENT

Not applicable.

## Data Availability

Not applicable.
